# Ion homeostasis and Na^+^ transport-related gene expression in two cotton (*Gossypium hirsutum* L.) varieties under saline, alkaline and saline-alkaline stresses

**DOI:** 10.1371/journal.pone.0256000

**Published:** 2021-08-10

**Authors:** Jialin Sun, Shuangnan Li, Huijuan Guo, Zhenan Hou

**Affiliations:** Department of Resources and Environmental Science, Shihezi University, Shihezi, Xinjiang, People’s Republic of China; Bhabha Atomic Research Centre, INDIA

## Abstract

The sensitivity of cotton to salt stress depends on the genotypes and salt types. Understanding the mechanism of ion homeostasis under different salt stresses is necessary to improve cotton performance under saline conditions. A pot experiment using three salt stresses saline stress (NaCl+Na_2_SO_4_), alkaline stress (Na_2_CO_3_+NaHCO_3_), and saline-alkaline stress (NaCl+Na_2_SO_4_+Na_2_CO_3_+NaHCO_3_) and two cotton varieties (salt-tolerant variety L24 and salt-sensitive variety G1) was conducted. The growth, ion concentrations, and Na^+^ transport-related gene expression in the cotton varieties were determined. The inhibitory effects of saline-alkaline stress on cotton growth were greater than that of either saline stress or alkaline stress alone. The root/shoot ratio under alkaline stress was significantly lower than that under saline stress. The salt-tolerant cotton variety had lower Na and higher K concentrations in the leaves, stems and roots than the salt-sensitive variety under different salt stresses. For the salt-sensitive cotton variety, saline stress significantly inhibited the absorption of P and the transport of P, K, and Mg, while alkaline stress and saline-alkaline stress significantly inhibited the uptake and transport of P, K, Ca, Mg, and Zn. Most of the elements in the salt-tolerant variety accumulated in the leaves and stems under different salt stresses. This indicated that the salt-tolerant variety had a stronger ion transport capacity than the salt-sensitive variety under saline conditions. Under alkaline stress and salt-alkaline stress, the relative expression levels of the genes *GhSOS*1, *GhNHX*1 and *GhAKT*1 in the salt-tolerant variety were significantly higher than that in the salt-sensitive variety. These results suggest that this salt-tolerant variety of cotton has an internal mechanism to maintain ionic homeostasis.

## 1. Introduction

Salt stress, which decreases crop yield and restricts the use of agricultural land, is a main threat to environmental resources and human health [1, 2]. It is estimated that more than 1000 million hectares of land is saline or sodic, and between 25% and 30% of irrigated lands (or about 70 million hectares) are salt-affected [3], which affects more than 20% of the total cultivated and 33% of irrigated agricultural lands [4]. In general, salt-affected soils can be divided into three categories: saline, alkaline, and saline-alkaline [5]. Globally, saline soils are the main salt-affected soils, amounting to 60% of the total salt-affected soils, while alkaline soils and saline-alkaline soils account for 26% and 14%, respectively [6]. Previous studies have shown that alkaline salt stress is quite different from neutral salt stress and these stresses should be called alkaline stress and saline stress, respectively [7]. Saline stress (NaCl and/or Na_2_SO_4_) consists of two main components including osmotic effect and ionic effect [8]. Alkaline stress (NaHCO_3_ and/or Na_2_CO_3_), as a result of its higher pH, can inhibit ion uptake and disrupt the ionic balance of plant cells and usually triggers more severe damage to plants than saline stress with a neutral pH [9, 10]. The effects of saline-alkaline stress include both saline stress caused by excessive salt ions and alkaline stress caused by high pH [11]. Therefore, understanding the response of plants to different salt stresses is essential for improving the salt tolerance of plants.

Cotton (*Gossypium hirsutum* L.) is one of the most important fiber crops and is moderately salt tolerant with a salinity threshold level of 7.7 dS m^-1^. Cotton can be used as a pioneer crop in salt-affected land due to its high salt tolerance [12]. However, its production can still be affected by adverse salt conditions [13]. Recently, Zhao et al. [14] proposed that the term “ionic imbalance” is more appropriate as excessive Na^+^ and Cl^-^ accumulation interferes with the influx or metabolism of other essential ions like K^+^ rather than causing nutrient toxicity per se. Not surprisingly, more than half of studies concerning salt stress focused on revealing the mechanisms of ion (mainly Na^+^ and K^+^) transport in plants [15]. Under NaCl stress, the Na concentration in the roots, stems and leaves of cotton significantly increases, whereas the K, Cu, B, and Mo concentrations in the roots, as well as the Mg and S concentrations in the leaves, significantly decreases [16]. This means that fertilizer recommendations in salt-affected field is highly challenging since nutrient application may increase or decrease plant salt tolerance, which may complicate the prediction of crop yield [17]. Although much attention has been given to salt tolerance in cotton, few studies have been conducted to show their different responses to saline and alkaline stresses [18].

Maintaining ion homeostasis by ion uptake and compartmentalization is one of the key mechanisms to improve the salt tolerance of plants [19]. Ionomics is a research method for qualitative as well as simultaneous quantitative analysis of elemental composition in plants by means of high-throughput analysis. Ionomics is important in understanding and appreciating the composition of elements and their role in physiological and biochemical functions and plant nutritional requirements [20]. All living organisms have developed efficient systems to acquire and store these elements and maintain their concentrations within a specific range that allows for normal development [21]. Maintaining Na^+^ homeostasis is important but not the only mechanism contributing to salt tolerance. The high external Na^+^ inhibits K^+^ uptake, and this often leads to low K^+^/Na^+^ ratios that disrupt cellular homeostasis [22]. Many genes have been identified to regulate the transport of Na^+^ and K^+^ in cotton. The *GhSOS1* gene encodes a plasma membrane Na^+^/H^+^ antiporter and is essential in regulating Na^+^ efflux at the cellular level. It also facilitates the long-distance transport of Na^+^ from the roots to the shoots [23]. The *GhNHX*1 gene, a vacuolar membrane-bound Na^+^/H^+^ antiporter that transports Na^+^ from the cytoplasm into the vacuole, is another effective method to minimize the Na^+^ concentration in the cytoplasm [24]. In addition, the K^+^ channel gene *GhAKT*1 controls and adjusts the absorption of K^+^ [25], which is also involved in regulating Na^+^ absorption and transport [26]. The abovementioned genes that regulate the K^+^/Na^+^ balance play crucial roles in the maintenance of K^+^/Na^+^ homeostasis in cotton under saline conditions.

Understanding the mechanism by which cotton maintains ion homeostasis under different salt stresses is of great agricultural importance, as soil salinity accounts for large losses in cotton yield worldwide. The sensitivity of cotton to salt stress depends on crop genotype and the type of salt [27]. We hypothesized that the abilities of salt-tolerant cotton varieties to maintain ion homeostasis (especially K^+^/Na^+^ homeostasis) were better than those of salt-sensitive cotton varieties under different salt stresses. In this study, we used two varieties of cotton that differ in their salt tolerance (salt-tolerant L24 and salt-sensitive G1). Cotton seedlings were subjected to saline stress (NaCl:Na_2_SO_4_ = 1:2), alkaline stress (Na_2_CO_3_:NaHCO_3_ = 1:2) or saline-alkaline stress (NaCl:Na_2_SO_4_:Na_2_CO_3_:NaHCO_3_ = 1:2:2:4). The contents of ions in the cotton tissues (leaves, stems, and roots) were investigated, and correlations between Na^+^ and other ion contents in the tissues of salt-tolerant and salt-sensitive cotton were analyzed. In addition, the expression levels of some genes related to K^+^/Na^+^ metabolism, such as *GhSOS*1, *GhNHX*1 and *GhAKT*1, were analyzed. The objectives of this study were to (i) clarify the response of ion contents to saline, alkaline and saline-alkaline stresses and (ii) compare the expression of the related K^+^/Na^+^-regulating genes between salt-tolerant and salt-sensitive cotton varieties.

## 2. Materials and methods

### 2.1 Experimental design

A pot experiment was performed in the greenhouse of the experimental station of Shihezi University, Shihezi, Xinjiang Province, China (44°18’N, 86°02’E). The soil used in the pot experiment was sampled from 0–30 cm topsoil from farmland at the experimental station. The soil at this site is a gray desert soil (Calcaric Fluvisol in the FAO/UNESCO System), and the texture is loam. Some of the soil physical and chemical properties were as follows: soil salinity (ECe, electrical conductivity of a saturated soil extract) 2.3 mS cm^-1^, pH 7.9, organic matter 14.9 g·kg^-1^, alkaline N 41.2 mg·kg^-1^, available P 10.6 mg·kg^-1^, and available K 248 mg·kg^-1^. The two cotton genotypes were Lumianyan No. 24 (a salt-tolerant cotton genotype, L24) and Ganmian No. 1 (a salt-sensitive cotton genotype, G1).

According to the salt components and pH in the majority of the salt-affected soils in Xinjiang, China, three categories of salt-affected soils (saline, alkaline, and saline-alkaline) were obtained by adding neutral salt (NaCl and Na_2_SO_4_) and/or alkaline salt (NaHCO_3_ and Na_2_CO_3_) to the sampled soil. The saline soil was treated by mixing NaCl and Na_2_SO_4_ at a 1:2 molar ratio. The alkaline soil was treated by mixing NaHCO_3_ and Na_2_CO_3_ at a 2:1 molar ratio. The saline-alkaline soil was treated by mixing NaCl, Na_2_SO_4_, NaHCO_3_, and Na_2_CO_3_ at a 1:2:4:2 molar ratio. In this experiment, seven treatments, including saline, alkaline, and saline-alkaline stresses, were set as follows: control (CK), low saline stress (S1), high saline stress (S2), low alkaline stress (A1), high alkaline stress (A2), low saline-alkaline stress (SA1), and high saline-alkaline stress (SA2). The soil ECe and pH values of the different salt stress treatments are shown in Table 1.

**Table 1:** The ECe and pH value of soil in different saline and alkaline stress treatments.

Treatment	ECe (mS·cm^-1^)	pH (H_2_O)
Control	2.3	7.9
Saline stress		
S1	6.65	8.05
S2	10.01	8.11
Alkaline stress		
A1	3.25	8.7
S2	3.95	9.5
Saline-alkaline stress		
SA1	6.65	8.7
SA2	10.01	9.5

Before beginning the experiment, the sampled soil was naturally dried, crushed, and sieved (2-mm pore size). According to the experimental design, neutral (NaCl and Na_2_SO_4_) and alkaline (NaHCO_3_ and Na_2_CO_3_) salts were distributed into solutions and sprayed onto the soil (the control treatment had the same volume of deionized water sprayed onto it). The amount of salt added to the sampled soil under different treatments was 0.286% (S1), 0.444% (S2), 0.094% (A1), 0.428% (A2), 0.482% (SA1), and 0.854% (SA2), respectively. After mixing evenly, the treated soil was left to stand for 1 month to ensure soil salt balance.

Plastic pots (sealed at the bottom) with an internal diameter of 15 cm and a height of 20 cm were used in this experiment. The treated soil was layered according to a soil bulk density of 1.25 g·cm^-3^ with 3.0 kg in each pot. The experiments followed a completely randomized block design with 6 replicates for each treatment. Cotton seeds were carefully selected, and 15 seeds were sown in each pot. Cotton seedlings were fixed at the 2-leaf stage, and 3 uniform seedlings were kept in each pot. During the experiment, the soil water content was kept at 60%-80% of the field capacity. The experiment was terminated 90 days after cotton emergence.

### 2.2 Determination of the biomass and growth inhibition rate of cotton

Six representative cotton plants were selected from each treatment. The plant samples were separated into the roots, stems, and leaves. Each plant component was washed with distilled water, dried in a forced air oven at 70°C for 72 h, and weighed. The growth inhibition rate (GIR) of cotton plants under saline, alkaline and saline-alkaline stress treatments was calculated as [28]:

$$GIR\left(\%\right)=\left(1-B_{T}/B_{CK}\right)\times 100\%$$ (1)

where *GIR* is the growth inhibition rate of plant tissue, *B*_*T*_ is the biomass of the treated plants, and *B*_*CK*_ is the biomass of the control plants.

### 2.3 Measurement of ion concentrations

The specific steps of plant ionomic analysis were as follows. First, samples were ground and 0.25 g of each sample was collected, 100 mg of which was added to 5 ml of 500 mM nitric acid (HNO_3_), followed by incubation for 24 h at room temperature and extraction at 85°C for 2 h; the resulting solution was then filtered. The ion concentrations (P, K, Na, Ca, Mg, Fe, Mn, Zn and Mo) in the roots, stems, and leaves were measured using inductively coupled plasma atomic emission spectrometry (ICP-AES; Thermo Fisher Scientific Inc., USA). The N concentration of the cotton plants was measured using an Auto-Kjeldahl Unit (B-339, Buchi Labortechnik AG, Switzerland). The relative contents of the elements N, P, K, Na, Ca, Mg, Fe, Mn, Zn and Mo in the cotton plants under saline, alkaline and saline-alkaline stress treatments were calculated as [29]:

$$RC=C_{T}/C_{CK}$$ (2)

where *RC* represents the relative element content, *C*_*T*_ represents the element concentration of the treated plant, and *C*_*CK*_ represents the element concentration of the control plant.

### 2.4 Analysis of *GhSOS*1, *GhNHX*1 and *GhAKT*1 gene expression

Four cotton leaves (the newest fully expanded leaves) were collected from each treatment group 90 days after cotton emergence. The samples were homogenized in liquid nitrogen before RNA isolation. The comparative *C*_T_ (ΔΔ*C*_T_) method [30] was used for quantitative analysis of relative gene expression in the cotton leaves. Total RNA was extracted with an RNA Extraction Kit (9767, Takara, Dalian, China), and cDNA was obtained with a reverse transcription kit (D6110A, Takara, Dalian, China). GAPDH was used as an internal reference gene. Specific primers were designed based on the nonconserved regions of the cotton genes (S1 Table). qRT-PCR amplification was performed to detect each gene. The Ct value (cycle threshold) of the target gene and the internal reference gene of the sample was repeated 3 times for each cDNA sample, and three cDNAs from different samples were used as biological replicates [31]. The specific method was as follows: the cotton leaves to be tested were reverse transcribed into cDNA after passing the test, total RNA was quantified, and the reaction mixture was added (S2 Table). The PCR system (20 μl) is shown in S3 Table.

### 2.5 Statistical analysis

The data were processed with Microsoft Excel 2010 software, and statistical analysis was performed with SPSS 19.0 software. Data were subjected to one-way analysis of variance (ANOVA) and comparisons using Tukey’s multiple comparison test at *P* < 0.05. Hierarchical cluster analysis and Na^+^ correlation analysis were carried out using the online website analysis software http://www.metaboanalyst.ca/.

## 3. Results

### 3.1 Biomass and growth inhibition rate

The effects of different salt stresses on cotton biomass are shown in Table 2. There was no significant difference in the total biomass of L24 (salt-tolerant) under low saline stress (S1) and low alkaline stress (A1) compared with CK, but the total biomass decreased by 17.3% and 10.3% under high saline stress (S2) and high alkaline stress (A2), respectively. The total biomass of L24 under low saline-alkaline stress (SA1) and high saline-alkaline stress (SA2) was 9.7% and 26.7% lower than that under CK. In contrast, the total biomass of G1 (salt-sensitive) decreased significantly with increasing soil salinity and pH. Compared with CK, the total biomass values of G1 under saline (S1, S2), alkaline (A1, A2), and saline-alkaline (SA1, SA2) stresses were reduced by 27.6–35.7%, 24.3–33.0%, and 43.4–87.7%, respectively.

**Table 2:** Biomass and growth inhibition rate of two cotton varieties under saline, alkaline and saline-alkaline stresses.

Treatment		Biomass (g∙plant-1)			Growth inhibition rate (%)		
		Shoot	Root	Total	Shoot	Root	Total
L24	CK	1.87±0.06 b	0.63±0.04 a	2.50±0.09 a	-	-	-
	S1	1.81±0.03 b	0.53±0.03 b	2.34±0.02 abc	3.0±1.3 b	15.9±4.2 c	6.3±0.6 cd
	S2	1.50±0.04 d	0.56±0.04 ab	2.07±0.03 d	19.6±2.2 a	10.6±5.6 c	17.3±1.3 b
	A1	2.04±0.07 a	0.43±0.05 c	2.43±0.03 ab	-7.0±3.5 c	31.7±8.4 b	2.7±1.0 d
	A2	1.75±0.09 bc	0.50±0.02 bc	2.24±0.13 cd	6.6±5.0 b	21.2±3.3 bc	10.3±3.8 c
	SA1	1.70±0.02 c	0.56±0.00 ab	2.26±0.02 bc	9.3±0.8 b	11.1±0.0 c	9.7±0.6 c
	SA2	1.64±0.11 cd	0.20±0.01 d	1.83±0.12 e	12.5±5.9 ab	68.8±0.9 a	26.7±4.6 a
G1	CK	1.57±0.03 a	0.42±0.00 a	1.99±0.03 a	-	-	-
	S1	1.06±0.03 c	0.38±0.01 b	1.44±0.04 b	32.4±2.1 d	9.8±1.4 e	27.6±2.0 d
	S2	0.94±0.02 d	0.33±0.03 c	1.28±0.05 c	39.8±1.5 c	20.3±7.8 cd	35.7±2.7 c
	A1	1.16±0.01 b	0.34±0.01 c	1.51±0.01 b	25.9±0.4 e	18.3±1.4 de	24.3±0.3 d
	A2	1.04±0.01 c	0.30±0.01 d	1.33±0.02 c	34.0±0.9 d	29.4±1.4 bc	33.0±0.9 c
	SA1	0.86±0.01 e	0.27±0.01 e	1.13±0.01 d	45.2±0.8 b	36.8±2.2 b	43.4±0.4 b
	SA2	0.60±0.03 f	0.22±0.01 f	0.82±0.03 e	62.0±1.9 a	46.5±2.1 a	58.7±1.7 a

Average data (±SE, n = 6) with the different lowercase letters in the same column indicate significant differences (*P*<0.05) among different saline and alkaline stress treatments.

In general, the growth inhibition rates of the total biomass in L24 were significantly lower than those in G1 under saline, alkaline, and saline-alkaline stresses (Table 2). The total inhibition rates of the two cotton varieties under saline-alkaline stress (SA1, SA2) were significantly higher than those under saline stress (S1, S2) and alkaline stress (A1, A2). The growth inhibition rates of the shoots and roots under saline, alkaline, and saline-alkaline stresses showed a different trend between L24 and G1 (Table 2). The shoot inhibition rates of L24 under saline and saline-alkaline stresses were significantly higher than that under alkaline stress, while the shoot inhibition rate of G1 followed the order saline-alkaline stress > saline stress > alkaline stress. There was no significant difference in the root inhibition rate of L24 between S1 and S2 or between A1 and A2. The root inhibition rate of G1 significantly increased as the soil salinity and the pH increased. The root inhibition rate of G1 increased from 9.8% to 20.3% as the soil salinity level increased from 6.65 dS m^-1^ (S1) to 10.01 dS m^-1^ (S2), and increased from 18.3% to 29.4% as the soil pH increased from 8.70 (A1) to 9.50 (A2). This indicated that there were significant differences in the responses of the two cotton varieties to different salt stresses.

### 3.2 Root/shoot ratio

**Fig 1:**
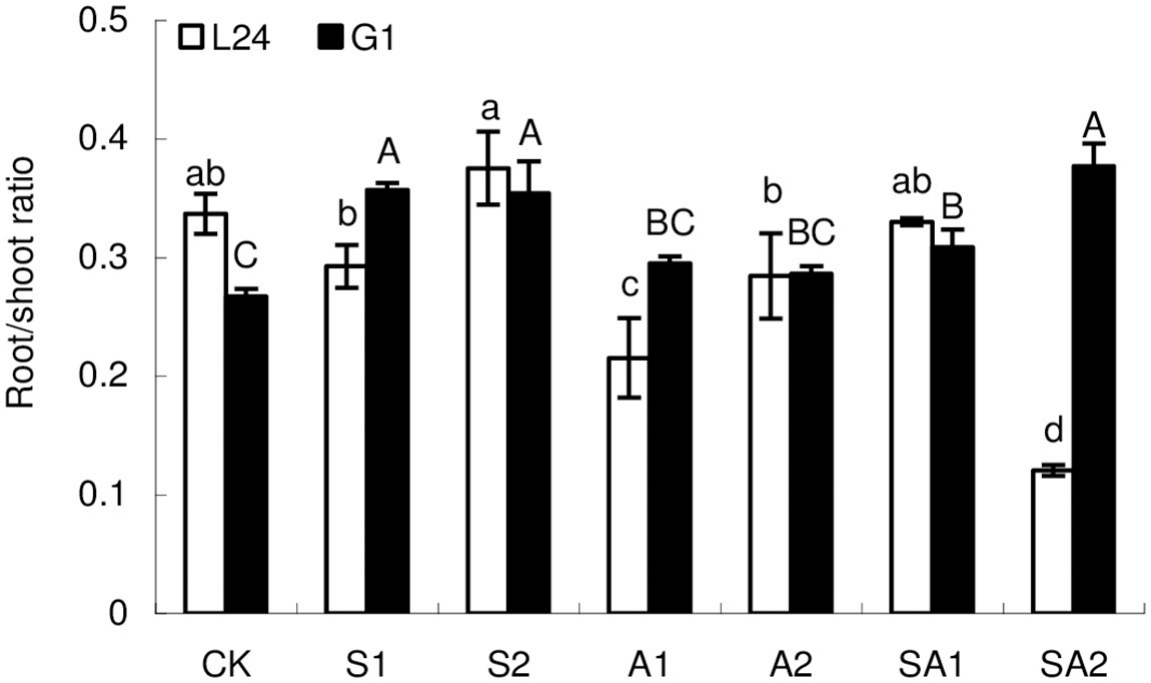
Root/shoot ratio of two cotton varieties under different salt stresses. Symbols indicate L24 cultivar (white bar) and G1 cultivar (black bar). Vertical bars represent ± standard error (n = 6). Bars labeled with the different lowercase letters or uppercase letters are significantly different (*P*<0.05) among different saline and alkaline stress treatments.

Under saline stress, there were no significant differences in the root/shoot ratio of L24 between the CK treatment and the S1 or S2 treatment, while the root/shoot ratio of S2 was 28.3% higher than that of S1 (Fig 1). Compared with the CK treatment, the root/shoot ratio of G1 in the S1 and S2 treatment groups increased by 32.2% and 31.1%, respectively. Under alkaline stress, the root/shoot ratio of L24 after A1 treatment decreased by 36.7% compared with that in the CK treatment, but there was no significant difference between the A2 treatment and the CK treatment. The root/shoot ratios of G1 after the A1 and A2 treatments were not different from CK treatment. Under saline-alkaline stress, the root/shoot ratio of L24 decreased by 64.6% in only the SA2 treatment group compared with CK treatment. In contrast, the root/shoot ratios of G1 after the SA1 and SA2 treatments were 14.3% and 39.7% higher than that in the CK treatment, respectively.

### 3.3 Ion contents of cotton under saline stress

**Fig 2:**
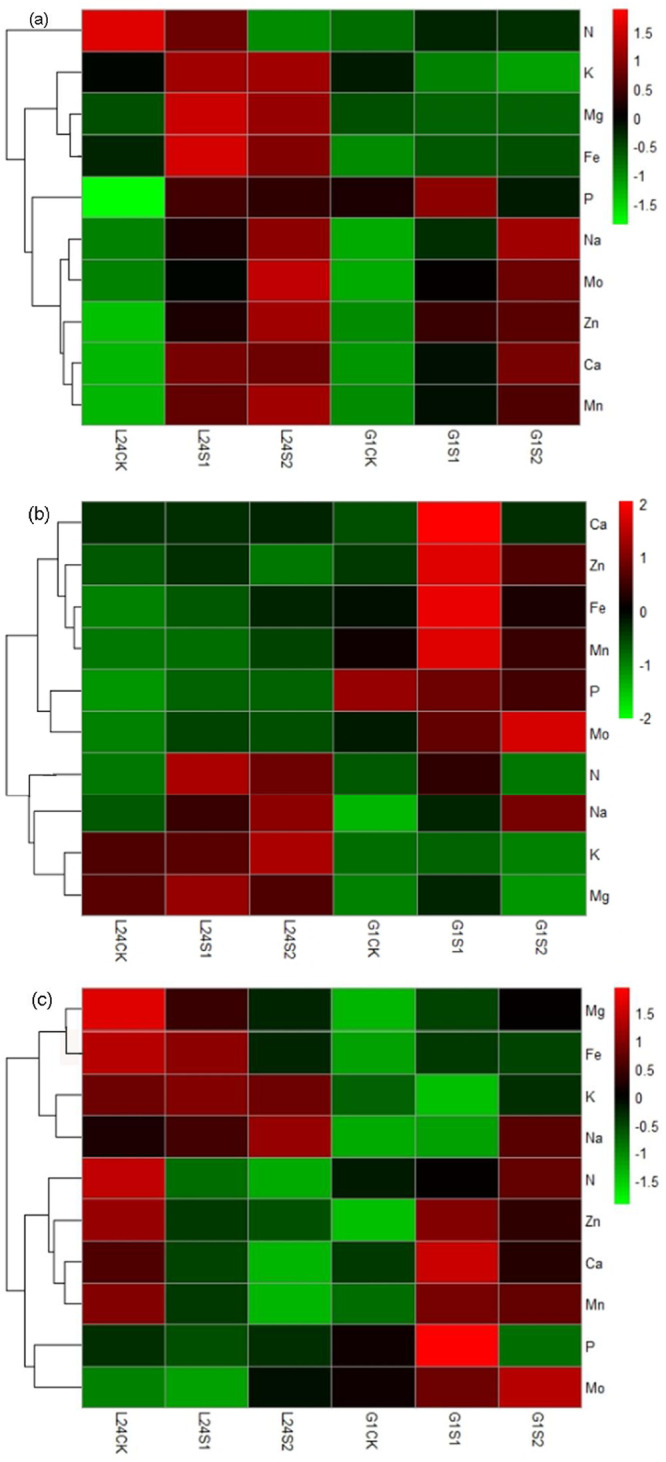
Hierarchical cluster analysis of element concentrations in leaves (a), stem (b), and root (c) of two cotton varieties (salt-tolerant variety L24, salt-sensitive variety G1) under saline stress (S1, S2). The abscissa represents the sample name, and the ordinate represents the elements. Different columns in the figure represent various samples, and rows represent element concentrations. The color represents the level of element concentrations in the sample.

To demonstrate the effects of saline stress on element distribution in cotton plants, we analyzed the concentrations of Na, N, P, K, Ca, Mg, Fe, Mn, Zn, and Mo in the leaves, stems and roots under saline stress (Table 3). In general, the Na concentration in cotton plant tissues increased significantly with increasing soil salinity. Compared with the CK treatment, the Na concentration in the leaves, stems and roots of salt-tolerant variety L24 increased by 2.3–4.0-fold, 1.1–1.8-fold, and 12.9%-43.7% under saline stress (S1 and S2), respectively, while the salt-sensitive variety G1 increased by 4.7–12.6-fold (leaves), 4.1–8.5-fold (stems) and 15.8%-290% (roots) under saline stress (S1 and S2), respectively. According to the element concentrations in the leaves, 10 elements could be divided into 2 groups (Fig 2a). The concentration of the first element (N) significantly decreased in L24 and increased in G1 under saline stress. The concentrations of K and P in the second group of elements significantly increased in L24 and decreased in G1. Other element concentrations (Mg, Fe, Zn, Ca, Mn) increased in both cotton varieties, and the concentration change in L24 was greater than that in G1. In the stems, the first group element concentrations (N, K, and Mg) increased under saline stresses (S1 and S2) in L24, while these same elements in G1 increased under S1 treatment and decreased under S2 treatment (Fig 2b). The increase in the second group element concentrations (Ca, Zn, Fe, Mn) in G1 under saline stress were higher than those in L24, and the P concentration decreased in G1 and increased in L24. In the roots, the element concentrations of N, Ca, Mg, Fe, Mn, and Zn increased in G1 under saline stress and decreased in L24 (Table 3, Fig 2c).

**Table 3:** The relative content of elements in leaves, stem and root of two cotton varieties under saline stress.

Treatment		Na	N	P	K	Ca	Mg	Fe	Mn	Zn	Mo
		Leaves									
L24	S1	3.33d	0.90b	1.76a	1.46a	1.42a	1.54a	2.40a	2.46b	1.74b	1.86c
	S2	5.01c	0.69c	1.73a	1.48a	1.41a	1.45a	1.95b	2.79a	2.21a	3.19b
G1	S1	5.71b	1.08a	1.16b	0.67b	1.17c	0.96b	1.72b	1.55d	1.57b	2.89b
	S2	13.63a	1.07a	0.92c	0.59b	1.35b	0.95b	1.98b	1.94c	1.67b	3.93a
		Stem									
L24	S1	2.11d	1.19a	1.11a	1.05c	0.99c	1.11a	1.22c	1.07c	1.14c	2.31b
	S2	2.77c	1.14ab	1.12a	1.33a	1.02bc	0.98b	1.48b	1.29b	0.92d	2.10b
G1	S1	5.10b	1.08b	0.94b	1.16b	1.73a	1.27a	1.88a	1.67a	1.79a	1.86c
	S2	9.52a	0.98c	0.88b	0.79d	1.06b	0.92c	1.15c	1.15bc	1.39b	2.60a
		Root									
L24	S1	1.13b	0.81c	0.97bc	1.05b	0.75c	0.83b	0.93b	0.77b	0.72c	0.91c
	S2	1.44b	0.78c	1.00b	1.01b	0.54d	0.73b	0.61c	0.59c	0.70c	1.35a
G1	S1	1.16b	1.02b	1.18a	0.47c	1.60a	1.20a	1.53a	1.42a	1.82a	1.20b
	S2	3.90a	1.09a	0.90c	1.33a	1.21b	1.31a	1.47a	1.37a	1.61b	1.36a

Different lowercase letters in the same column for the roots, stems, or leaves indicate significant differences (*P*<0.05) between individual treatments.

**Fig 3:**
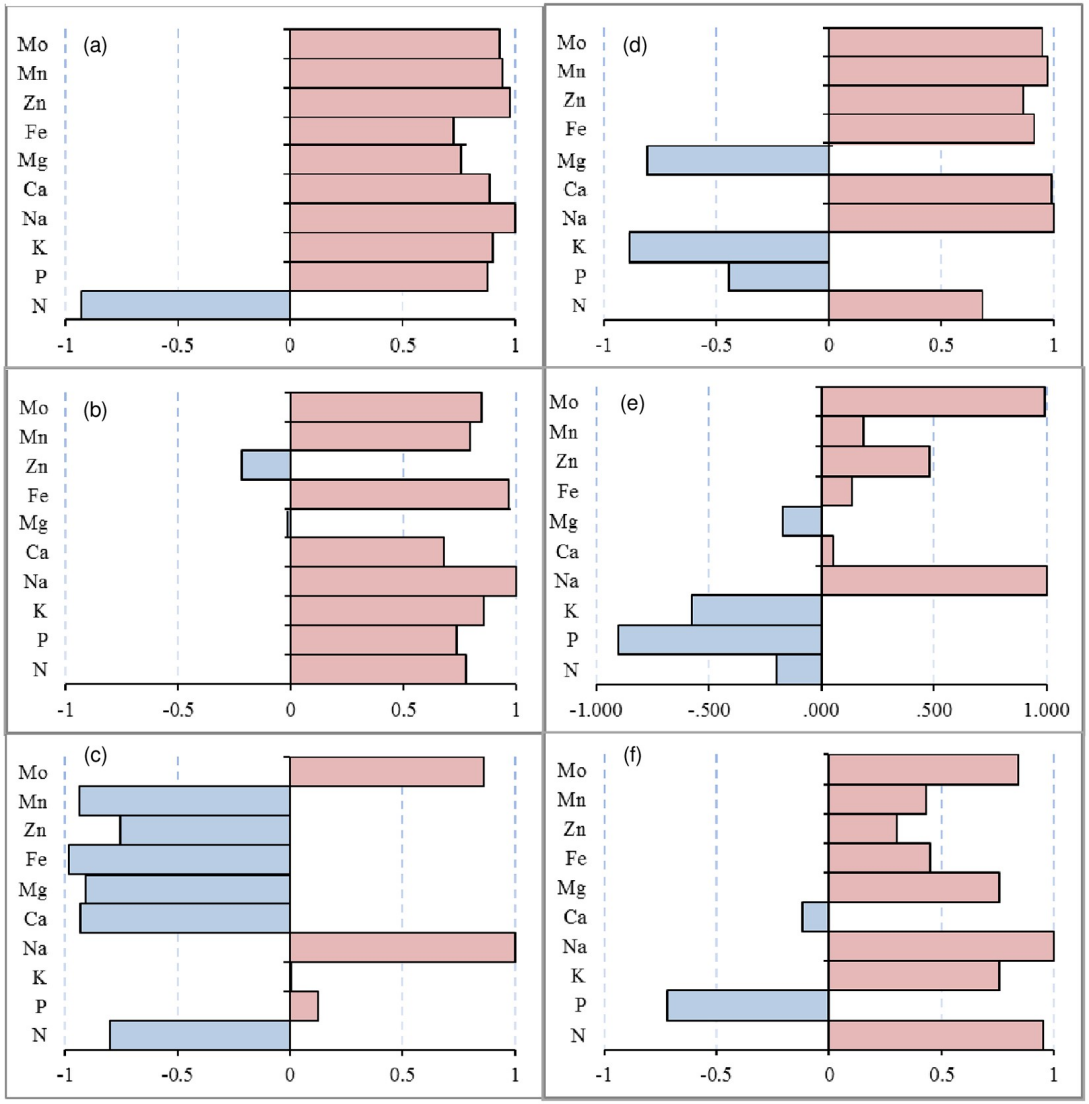
Correlation between the concentration of Na and other elements in leaves, stem, and root of two cotton varieties (salt-tolerant variety L24, salt-sensitive variety G1) under saline stress. (a), (b), and (c) stand for the leaves, stem, and root of L24, respectively; (d), (e), and (f) stand for the leaves, stem, and root of G1, respectively.

Salt stress is mainly caused by excessive amounts of Na^+^ ions, and thus, it is essential to understand the correlation between Na^+^ ions and other elements. Therefore, the correlation between Na and other elements in the leaves, stems, and roots under saline stress was analyzed (Fig 3). In the leaves, Na was significantly negatively correlated with only N in L24 under saline stress (Fig 3a) but had a negative correlation with K, Mg and P in G1 (Fig 3d). In the stems, Na was negatively correlated with Zn and Mg in L24 (Fig 3b) but had a negative correlation with N, P, K, and Mg in G1. Among them, the P and K concentrations were significantly negatively correlated with Na in G1 (Fig 3e). In the roots, Na was significantly positively correlated with most of the elements except for P, K and Mo in L24 (Fig 3c), and P was only significantly negatively correlated with Na in G1 (Fig 3f). The data showed that there were more elements negatively correlated with Na in the leaves and stems of the salt-sensitive variety (G1) than in the salt-tolerant variety (L24). In the roots, the salt-tolerant variety had more elements that were negatively correlated with Na than the salt-sensitive variety.

### 3.4 Ion contents of cotton under alkaline stress

Knowing the relative content of ions (Table 4), compared with the CK treatment, the Na concentration of salt-tolerant cultivar L24 and salt-sensitive cultivar G1 in the leaves increased by 1.0-, 1.74-, and 0.29-, 22.8-fold; in the stems, these values increased by 0.78-, 1.57- and 3.4-, 10.1-fold after the A1 and A2 treatments, respectively. In the roots, compared with the CK treatment, the Na concentration of L24 decreased by 12.9% after A1 treatment and increased by 16.2% after A2 treatment; the Na concentration in G1 increased by 31.8% and 38.1% after the A1 and A2 treatments, respectively.

**Table 4:** The relative content of elements in leaves, stem and root of two cotton varieties under alkaline stress.

Treatment		Na	N	P	K	Ca	Mg	Fe	Mn	Zn	Mo
		Leaves									
L24	A1	2.03c	0.81bc	1.85a	1.48b	1.23b	1.33b	2.59b	2.12b	2.25a	1.58c
	A2	2.74b	0.68c	1.67b	1.63a	1.30a	1.42a	2.06c	2.09b	1.87b	2.69b
G1	A1	1.29d	0.98ab	0.93c	0.94c	0.97c	0.83c	0.64d	0.71c	0.91c	1.53c
	A2	23.8a	1.10a	0.86d	0.74d	0.63d	0.77d	3.03a	2.30a	0.64d	3.94a
		Stem									
L24	A1	1.78d	1.11ab	1.26b	1.09a	1.03b	0.94a	2.21a	1.49a	1.13b	1.68b
	A2	2.57c	1.19a	1.41a	1.05ab	1.13a	0.98a	1.74b	1.55a	1.20b	3.88a
G1	A1	4.39b	0.88c	1.10c	1.02bc	1.09a	0.95a	0.50d	0.92c	1.41a	1.15c
	A2	11.1a	1.06b	0.81d	0.91c	0.83c	0.97a	0.98c	1.15b	0.85c	1.57b
		Root									
L24	A1	0.87c	0.85b	0.90b	1.00b	0.79c	0.74b	0.84b	0.81a	0.74b	0.81c
	A2	1.16b	0.80b	0.90b	1.10a	0.84b	0.68c	0.93a	0.80a	0.75b	1.12b
G1	A1	1.32a	1.08a	1.01a	0.74c	1.01a	0.86a	0.62c	0.74b	1.61a	1.28a
	A2	1.38a	0.98a	0.85b	0.80b	0.99a	0.73b	0.56d	0.52c	0.61c	1.32a

Different lowercase letters in the same column for the roots, stems, or leaves indicate significant differences (*P*<0.05) between individual treatments.

**Fig 4:**
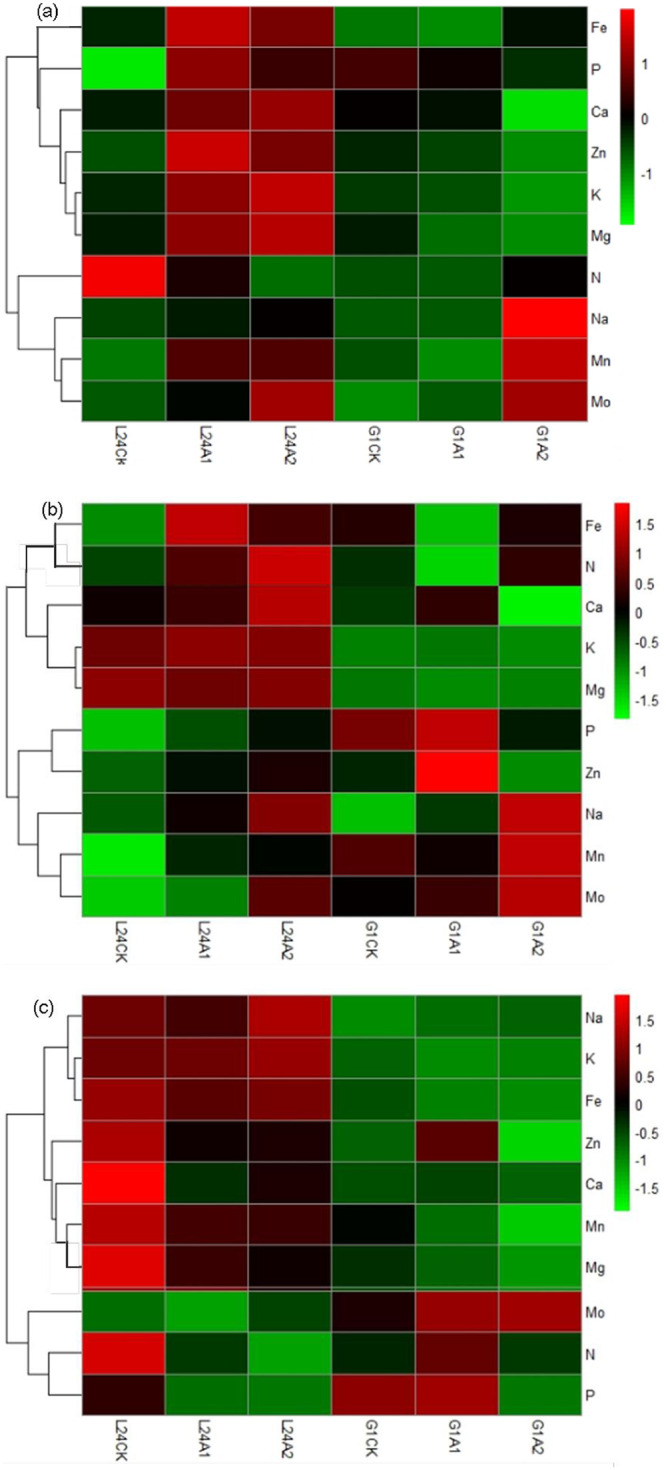
Hierarchical cluster analysis of element concentrations in leaves (a), stem (b), and root (c) of two cotton varieties (salt-tolerant variety L24, salt-sensitive variety G1) under alkaline stress (A1, A2). The abscissa represents the sample name, and the ordinate represents the elements. Different columns in the figure represent various samples, and rows represent element concentrations. The color represents the level of element concentrations in the sample.

According to the hierarchical cluster analysis of the ion group changes of the two cotton varieties, the nutrient elements in each organ were roughly divided into two groups under alkaline stress (Fig 4). In the leaves, the concentration of the first group of elements (P, Ca, Zn, K, and Mg) increased in L24 and decreased in G1. In the second group elements, Mn and Mo increased significantly in both cotton varieties, and the N content significantly decreased in L24 and increased in G1 (Fig 4a). In the stems, the concentrations of the first group of elements (Fe, N, Ca, and K) increased in L24 and were significantly higher than those in G1. The concentrations of the second group of elements (P, Zn, Mn and Mo) also increased in L24, and the concentrations of P and Zn decreased significantly in G1 after the A2 treatment (Fig 4b). Compared with CK, the concentrations of most elements in the roots were significantly reduced in both varieties (Table 4). The relative contents of the first group of elements (K, Fe and Mn) in L24 were significantly higher than those in G1, while the relative contents of the second group elements (Mo, N, P) were significantly lower than those in G1 (Fig 4c).

**Fig 5:**
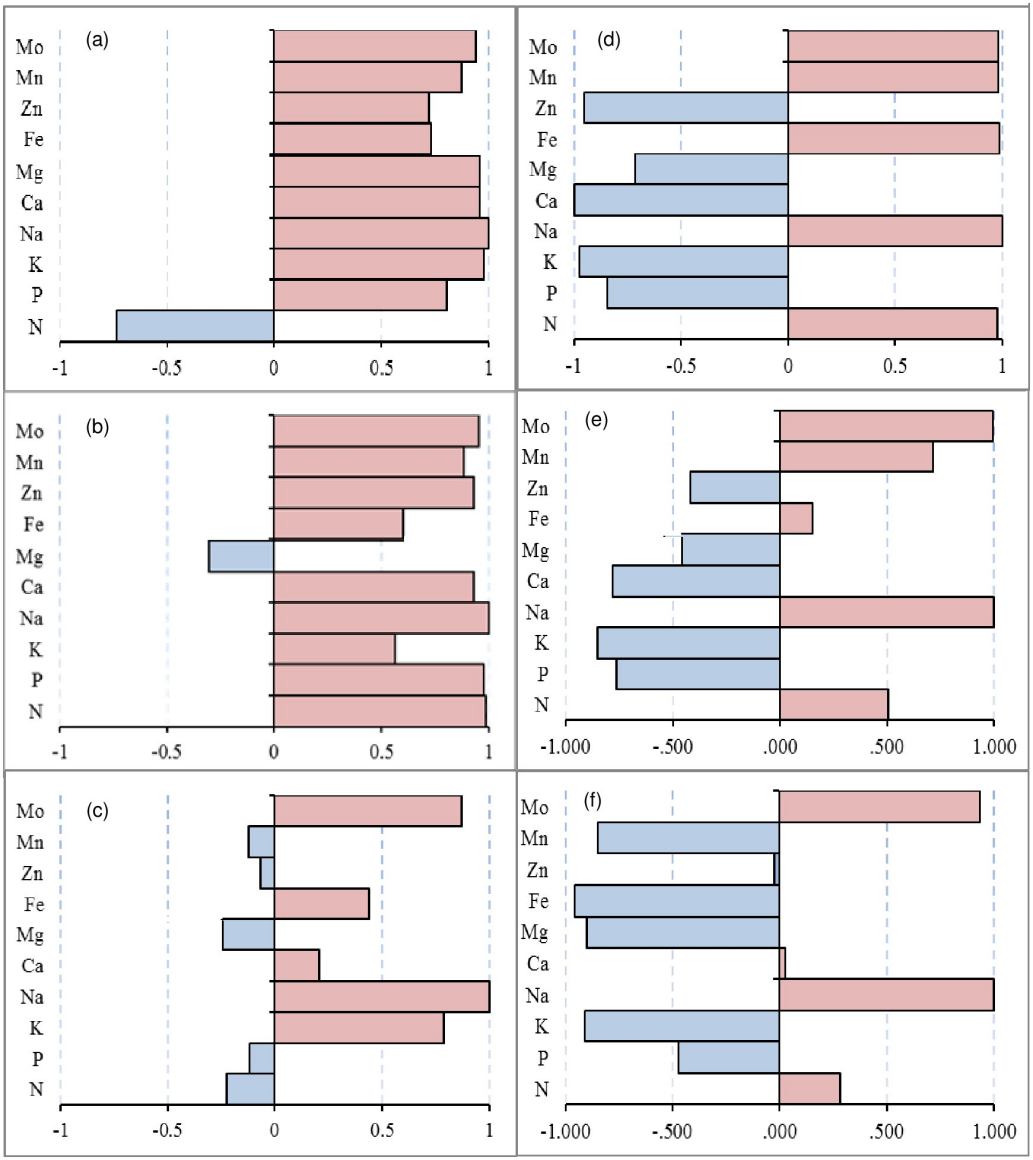
Correlation between the concentration of Na and other elements in leaves, stem, and root of two cotton varieties (salt-tolerant variety L24, salt-sensitive variety G1) under alkaline stress. (a), (b), and (c) stand for the leaves, stem, and root of L24, respectively; (d), (e), and (f) stand for the leaves, stem, and root of G1, respectively.

In the leaves, the Na levels were significantly negatively correlated with N in L24 under alkaline stress (Fig 5a) and significantly negatively correlated with P, K, Ca, Mg and Zn in G1 (Fig 5d). In the stems, Na levels were significantly positively correlated with nearly all elements except for Mg in L24 (Fig 5b) and were negatively correlated with P, K, Ca, Mg, and Zn in G1, where P, K, and Ca were significantly negatively correlated with Na (Fig 5e). In the roots, the Na levels were negatively correlated with N, P, Mg, Zn and Mn in L24, among which only Al was significantly negatively correlated with Na (Fig 5c). The Na concentration in G1 was significantly negatively correlated with K, Mg, Fe and Mn (Fig 5f).

### 3.5 Ion contents in cotton under saline-alkaline stress

**Fig 6:**
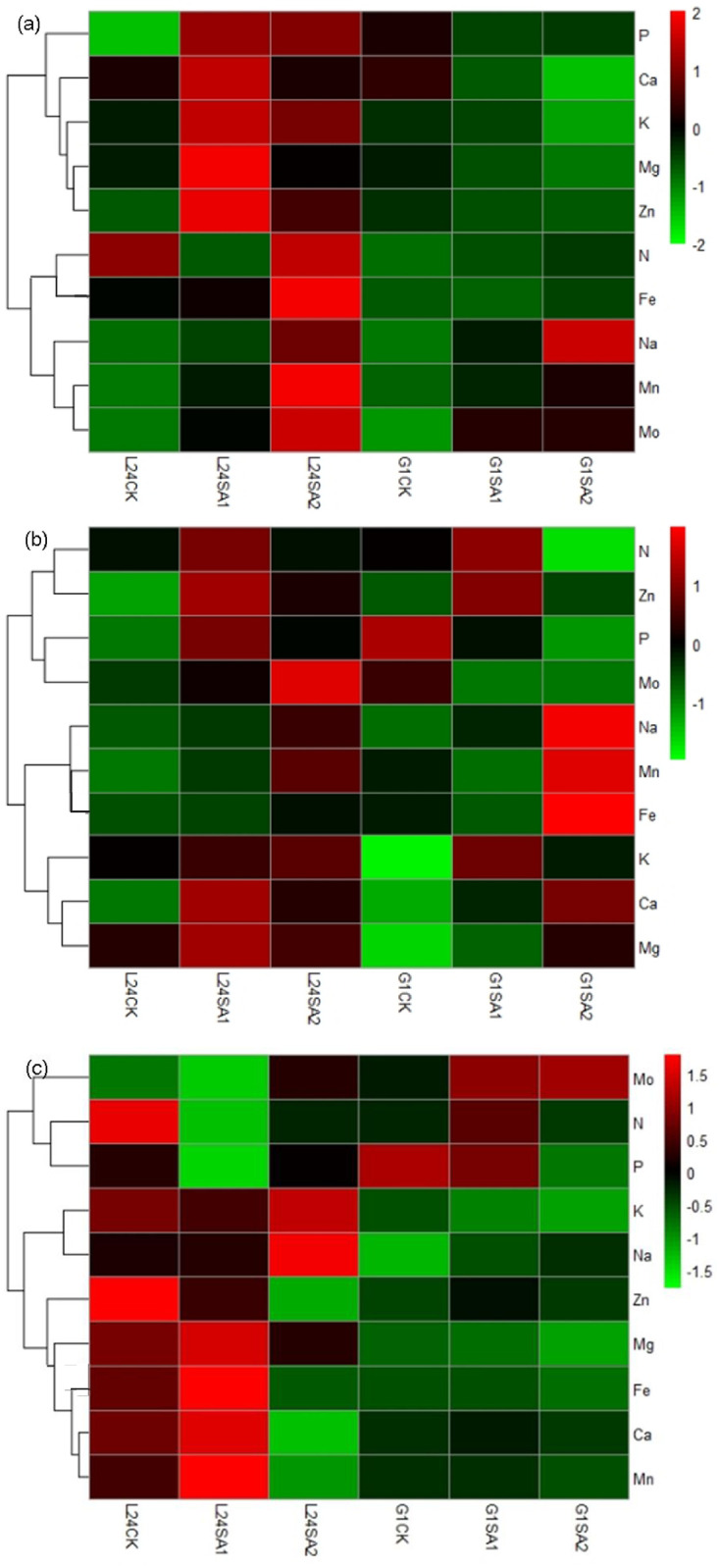
Hierarchical cluster analysis of element concentrations in leaves (a), stem (b), and root (c) of two cotton varieties (salt-tolerant variety L24, salt-sensitive variety G1) under saline-alkaline stress (SA1, SA2). The abscissa represents the sample name, and the ordinate represents the elements. Different columns in the figure represent various samples, and rows represent element concentrations. The color represents the level of element concentrations in the sample.

Under saline-alkaline stress, the Na concentrations in the cotton samples increased significantly with increasing stress level. Compared with CK treatment, the Na concentration of salt-tolerant cultivar L24 and salt-sensitive cultivar G1 increased by 1.9-, 9.1-fold and 10.6-, 35.8-fold in the leaves; 1.2-, 5.0-fold and 9.7-, 44.1-fold in the stems; and 1.9%, 69.8% and 108.3%, 142.5% in the roots after SA1 and SA2 treatment, respectively (Table 5). There were obvious differences in the changes in ionic groups in the different organs of the two cotton varieties (Fig 6). The 10 elements can be divided into 2 groups according to the characteristics of their content changes. In the leaves, the concentrations of the first group of elements (P, Ca, K, Mg, Zn) increased in L24 and decreased significantly in G1 under saline-alkaline stress (Fig 6a). The second group of elements (N, Fe, Mn, and Mo) increased with increasing stress in both L24 and G1. In the stems, only the Zn concentration in the first group of elements increased in both varieties, and the concentrations of the other elements (N, P, Mo) increased in L24 and decreased in G1 (Fig 6b). The concentrations of the second group of elements (Mn, Fe, K, Ca, Mg) increased in both varieties. In the roots, the concentrations of the first group elements (Mo, N, P) after SA2 treatment were higher than those after SA1 treatment in L24, but the opposite was true in G1 (Fig 6c). The concentrations of the second group of elements (Zn, Mg, Fe, Ca, Mn) after SA1 treatment were higher than those after SA2 treatment in both varieties.

**Table 5:** The relative content of elements in leaves, stem and root of two cotton varieties under saline-alkaline stress.

Treatment		Na	N	P	K	Ca	Mg	Fe	Mn	Zn	Mo
		Leaves									
L24	SA1	2.86d	0.74b	2.08a	1.49a	1.31a	1.42a	1.12c	1.86b	2.44a	2.14c
	SA2	10.1c	1.06a	2.00a	1.32b	1.01b	1.04b	2.95a	4.50a	1.70b	4.38a
G1	SA1	11.6b	1.04a	0.83b	0.96c	0.76c	0.92c	0.78d	1.51b	0.88c	3.79b
	SA2	36.8a	1.08a	0.85b	0.74d	0.57d	0.86c	1.27b	2.03b	0.83c	3.91b
		Stem									
L24	SA1	2.17d	1.17a	1.59a	1.14c	1.31a	1.19c	1.17b	1.43b	1.49a	2.39b
	SA2	5.98c	0.99b	1.27b	1.21c	1.17b	1.04d	1.77a	2.63a	1.28b	5.97a
G1	SA1	10.6b	1.18a	0.73c	3.91a	1.15b	1.30b	0.57c	0.60c	1.30a	0.81c
	SA2	45.1a	0.70c	0.54d	2.78b	1.33a	1.66a	2.97a	2.09a	1.03b	0.71c
		Root									
L24	SA1	1.02d	0.79d	0.90b	0.88b	1.17a	1.19a	1.52a	1.54a	0.71b	0.68b
	SA2	1.70c	0.87c	0.99a	1.21a	0.54c	0.85c	0.33d	0.39c	0.40c	1.68a
G1	SA1	2.08b	1.07a	0.98a	0.69c	1.03b	0.96b	0.97b	0.97b	1.14a	1.47a
	SA2	2.42a	0.99b	0.89b	0.48d	0.97b	0.80c	0.75c	0.84b	1.04a	1.44a

Different lowercase letters in the same column for the roots, stems, or leaves indicate significant differences (*P*<0.05) between individual treatments.

**Fig 7:**
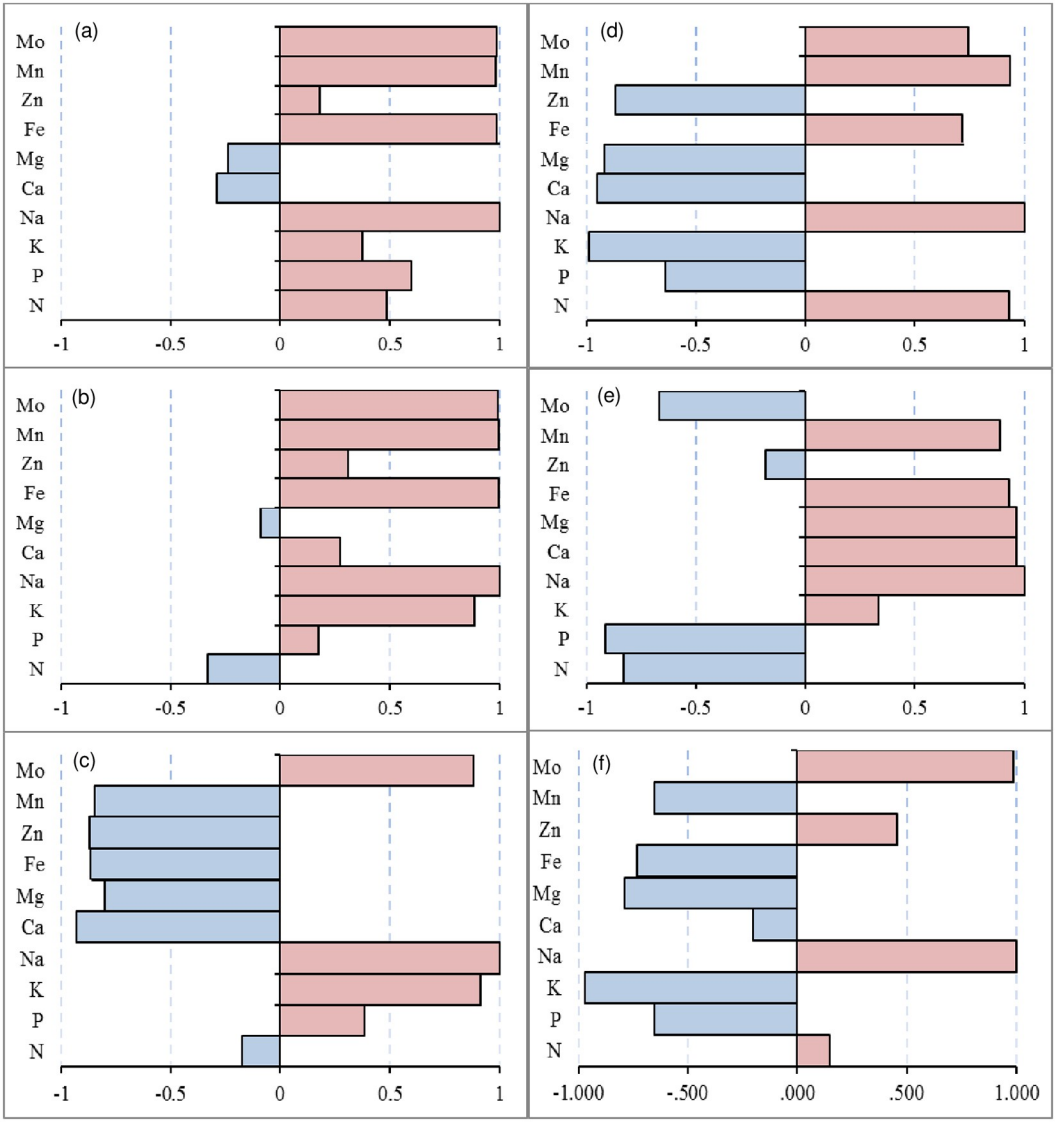
Correlation between the concentration of Na and other elements in leaves, stem, and root of two cotton varieties (salt-tolerant variety L24, salt-sensitive variety G1) under saline-alkaline stress. (a), (b), and (c) stand for the leaves, stem, and root of L24, respectively; (d), (e), and (f) stand for the leaves, stem, and root of G1, respectively.

Under saline-alkaline stress, Na levels in the leaves were only negatively correlated with Ca and Mg in L24 (Fig 7a) and were significantly negatively correlated with P, K, Ca, Mg, and Zn in G1 (Fig 7d). In the stems, the Na levels in L24 were negatively correlated with N and Mg (Fig 7b) and were significantly negatively correlated with N, P and Mo in G1 (Fig 7e). The content of Na in the roots was negatively correlated with Ca, Mg, Fe, Zn and Mn in L24 (Fig 7c) and negatively correlated with P, K, Mg, Mn and Fe in G1 (Fig 7f).

### 3.6 Expression of the genes *GhSOS*1, *GhNHX*1 and *GhAKT*1

**Fig 8:**
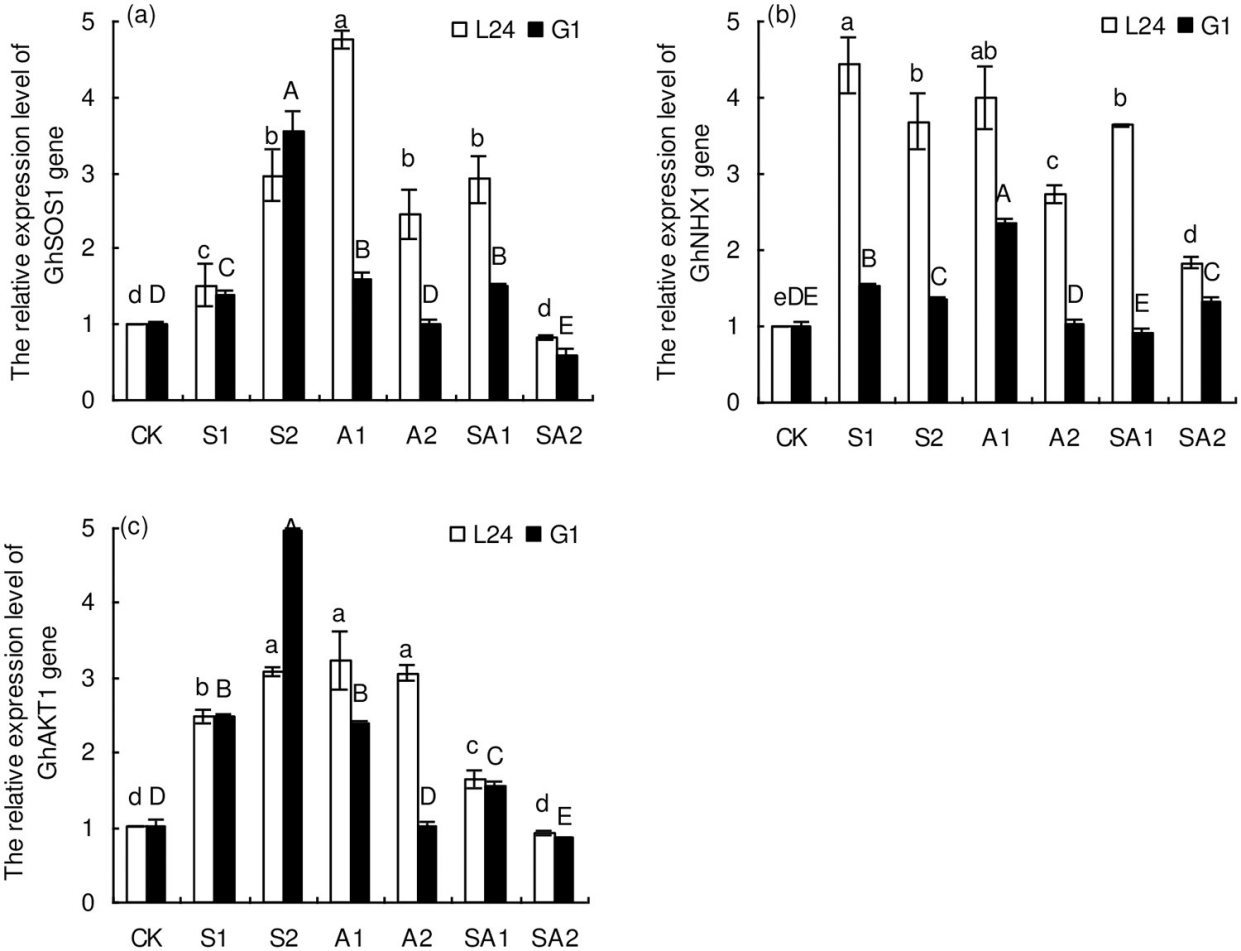
Relative expression of *GhSOS*1 (a), *GhNHX*1 (b) and *GhAKT*1 (c) genes of two cotton varieties (salt-tolerant variety L24, salt-sensitive variety G1) under different salt stresses. Symbols indicate L24 cultivar (white bar) and G1 cultivar (black bar). Vertical bars represent ± standard error (n = 4). Bars labeled with the different lowercase letters or uppercase letters are significantly different (*P*<0.05) among different saline and alkaline stress treatments.

The expression of the *GhSOS*1 gene in the salt-tolerant variety (L24) and salt-sensitive variety (G1) increased significantly with increasing soil salinity (Fig 8a). Compared with CK, the expression of the *GhSOS*1 gene increased by 51% (S1) and 195% (S2) in L24 and by 39% (S1) and 255% (S2) in G1. Under alkaline stress, the expression of the *GhSOS*1 gene in L24 treated with A1 and A2 was 3.73 and 1.44 times higher than that in CK, respectively. The *GhSOS*1 gene expression of G1 was 61% higher after A1 treatment than after CK treatment, and there was no significant difference between the A2 and CK treatments. Under saline-alkaline stress, the *GhSOS*1 gene expression of L24 was 1.90-fold higher in the SA1 treatment group than in the CK, and there was no significant difference between the SA2 and CK treatments. The expression of the *GhSOS1* gene in G1 showed a significant increase after SA1 treatment compared with CK, while its expression after SA2 treatment decreased significantly. In summary, the expression of *GhSOS*1 in L24 was significantly higher than that in G1 under alkaline stress and saline-alkaline stress.

Compared with CK, the relative expression of the *GhNHX*1 gene after S1 and S2 treatments increased by 3.44- and 2.68-fold in L24 and increased by 52% and 35% in G1, respectively (Fig 8b). Under alkaline stress, the expression of the *GhNHX*1 gene in L24 was 2.99- and 1.74-fold higher in the A1 and A2 treatment groups than in the CK, respectively, while the *GhNHX*1 gene expression in G1 was significantly increased only after A1 treatment. Under saline-alkaline stress (SA1, SA2), the relative expression levels of the *GhNHX*1 gene in L24 significantly increased compared with that in CK, while the *GhNHX*1 gene expression in G1 was significantly increased after only SA2 treatment. Generally, the expression of *GhNHX*1 in L24 was significantly higher than that in G1 under saline, alkaline and saline-alkaline stresses.

Under saline stress, the expression levels of *GhAKT*1 in both cotton varieties increased significantly with increasing soil salinity (Fig 8c). Compared with CK, the expression of the *GhAKT*1 gene in L24 and G1 increased by 1.45- and 2.06-fold and 1.46- and 3.95-fold after the S1 and S2 treatments, respectively. *GhAKT*1 expression levels in L24 under the A1 and A2 treatments were significantly higher than that of CK, and there was no significant difference between the A1 and A2 treatments in L24. In contrast, the expression level of *GhAKT*1 in G1 was significantly increased only after A1 treatment. Under saline-alkaline stress, *GhAKT*1 gene expression in L24 was 62% higher in the SA1 treatment than in the CK, and there was no significant difference between the SA2 and CK treatments. *GhAKT*1 gene expression in L24 under SA1 treatment was 54% higher than that under CK but was reduced by 15% under SA2 treatment compared with CK. Overall, the expression of *GhAKT*1 in L24 was significantly higher than that in G1 under alkaline stress and saline-alkaline stress.

## 4. Discussion

Soil salinization negatively impacts agricultural production throughout the world, and its impact is increasing [32]. The base ion contents of saline-alkali soils are excessively high, which hinders crop growth and causes damage, thereby reducing yields and constraining agricultural development [33]. Inhibition of the vegetative development of the shoots and roots is the primary response to salt stress [34]. It is commonly known that in a large variety of plants, shoot growth is more sensitive to salinity than the roots, and the root/shoot ratio therefore typically increases under salt stress [35]. However, there are few studies on the differences in cotton growth under saline stress, alkaline stress and saline-alkaline stress. Guo et al. [36] showed that the relative biomass of cotton varieties with different salt tolerance decreased with the increase of saline and alkali stress, and the relative biomass of leaves of salt tolerant varieties was significantly higher than that of salt sensitive varieties. Our results show that, from the perspective of varieties, the relative biomass of L24 above and below ground is significantly higher than that of G1, indicating that the growth inhibition rate of G1 under salt-alkali stress is significantly higher than that of L24. At the same time, the effects of saline-alkaline stress on the growth inhibition rates of total biomass in two cotton varieties (salt-tolerant and salt-sensitive) were significantly greater than that of saline stress or alkaline stress (Table 2). Previous studies also showed that mixed saline-alkaline stress is more severe than either saline or alkaline stress alone [37, 38]. In addition, we found that the inhibition of alkaline stress on the root growth of the two cotton varieties was greater than that of saline stress, and the root/shoot ratio of cotton under alkaline stress was significantly lower than that under saline stress (Fig 1). Under saline-alkaline stress, the root/shoot ratio of the salt-tolerant variety (L24) decreased, while that of the salt-sensitive variety (G1) increased significantly.

Salt stress can annihilate the ion homeostasis of plant cells, destroy the ionic balance, and affect the distributions of Na^+^, K^+^, Ca^2+^ in cells [10]. K^+^/Na^+^ homeostasis is the primary core response for plants to tolerate salinity [39]. Our results showed that the Na concentration in the leaves, stems, and roots of cotton increased significantly with increasing stress level and that the increase in Na concentration was higher under saline-alkaline stress than under saline stress or alkaline stress alone. Guo et al. [40] also observed that saline and alkaline stresses increased the Na concentration of cotton plants, but the increase in Na concentration was higher in the leaves, stems, and roots under alkaline stress than under saline stress. We found that the Na concentration of the salt-tolerant variety was significantly lower than that of the salt-sensitive variety under saline, alkaline, and saline-alkaline stresses. Indicating that cotton plants tolerated salt stress by storing Na in the leaves. In addition, the concentrations of Na and K in the salt-tolerant variety increased with increasing salinity, while the Na concentration increased and the K concentration decreased in the salt-sensitive variety. This indicated that the salt-tolerant variety has a strong ability to regulate Na^+^ and K^+^ absorption and transport. It was also found that the Na content of salt-sensitive varieties was significantly higher under alkali stress than under saline stress, corresponding to the result that the root inhibition rate of salt-sensitive varieties was greater than that of salt-tolerant varieties under alkali stress. Exposure to saline growth conditions leads to novel detection of OU-sensitive, Na^+^ /K^+^ ATPases in the plasma membrane of sunflower seedling root meristem cells [41]. Calcium significantly inhibits the expression of oubain-sensitive ATPases. Application of Ca^2+^ (10 mM) has been reported to inhibit OU-sensitive ATPases in salt stressed root cells of sunflower semi salt-tolerant variety (KBSH 53) [42]. This observation indicated that the accumulation of K^+^ and Ca^2+^ was a mechanism to buffer the increase of Na^+^ concentration in salt tolerant varieties. Chen et al. [43] showed that both salt and alkali stress decreased the K^+^ concentration and increased the Na^+^ concentration to a greater degree under mixed salt-alkali stress than under salt stress alone, indicating that Na^+^ competes with K^+^ for uptake during salt-alkali stress. Yang et al. [10] reported that the concentrations of Na^+^ and K^+^ both increased with increasing salinity, which implies that there was no competitive inhibition of absorption in *Kochia sieversiana*.

Salt stress not only inhibited the uptake of macroelements (N, P, K, Ca, Mg, S) by crops but also limited the uptake of trace elements (Fe, Cu, Zn, Mn, B, etc.), resulting in nutrient deficiency and cell metabolic disorder [44]. To mitigate the negative effects of salinity on plant productivity, it is necessary to understand these nutritional disorders. Under neutral salt stress, changes in the concentrations of inorganic ions have been reported by many researchers [27]. In the present study, the changes in 9 related elements (N, P, K, Ca, Mg, Fe, Mn, Zn, Mo) in two cotton varieties (salt-tolerant and salt-sensitive) under saline, alkaline and saline-alkaline stresses were examined. Overall, the Mo concentration in the leaves, stems, and roots of the two cotton varieties increased significantly and was positively correlated with the Na concentration under different salt stresses. This is in agreement with the results of a previous study [40]. Under saline stress, ionic imbalance restricts nutrient access and transport within plants, resulting in reduced concentrations of Mg, Ca, P and N in the roots and leaves [27]. We found that saline stress significantly increased the concentration of most elements in the leaves and stems of the salt-tolerant variety (only the concentration of N in the leaves and Zn in the stems decreased), while the concentrations of P, K and Mg in the leaves and N, P, K, and Mg in the stems of the salt-sensitive variety decreased. In the roots, most element concentrations decreased in the salt-tolerant variety, but most element concentrations increased in the salt-sensitive variety (only the P concentration decreased) (Table 3, Fig 2). This indicated that the salt-tolerant variety had a stronger mineral element transport capacity than the salt-sensitive variety under saline stress. There were significant differences in ion changes of cotton varieties with different salt tolerance under saline stress. Our findings are similar to those of Guo et al. [16], who reported that salt-sensitive varieties accumulated mineral elements in the roots, whereas salt-tolerant varieties transported mineral elements to the leaves under NaCl stress. However, some findings have reported that NaCl treatment reduced the K, Ca and Mg concentrations in cotton leaves and roots, and there were no significant differences between two cotton cultivars (salt-tolerant CCRI-79 and salt-sensitive Simian 3) in the variation of ion content under NaCl stress [15].

Alkaline stress mainly results from the levels of the alkaline salts NaHCO_3_ and Na_2_CO_3_ in soil, which can greatly affect the absorption of cations and inorganic anions and can also disrupt the ionic balance of the tissues [45]. However, the responses of ions and their interactions to alkaline stress have not yet been fully elucidated [46]. Alkaline soil will limit the absorption of nutrients by plants, and trace elements such as Fe, Zn, and Mn cannot be used by plants in alkaline soil [47]. However, Guo et al. [45] found that alkaline stress reduced the NO_3_^-^, H_2_PO_4_^-^ and SO_4_^2-^ contents in the leaves of cotton but had no significant effects on the Ca, Mg, Fe, Cu, Zn, and Mn contents in leaves. Wang et al. [48] reported that the contents of NO_3_^−^, H_2_PO_4_^−^ and SO_4_^2-^ in rice shoots under alkaline stress were lower than those under saline stress. Our results showed that the concentrations of P, K, Ca, Mg, and Zn in the leaves and P, K, Mg, Fe, and Mn in the roots of the salt-sensitive variety decreased under both alkaline and saline-alkaline stress (Tables 4 and 5; Figs 4 and 6). This result indicated that the inhibition of ion absorption and transport of salt-sensitive varieties was greater under alkaline stress and saline-alkaline stress than under saline stress. Guo et al. [40] also showed that the inhibition of ion absorption was greater under alkali stress than under salt stress. In addition, we found that the variation of ion concentration of the salt-tolerant variety under alkaline stress and saline-alkaline stress was similar to that under saline stress. The concentration of most elements increased in the leaves (only the N content decreased) and decreased in the roots (including N, P, Ca, Mg, Fe, Mn, Zn) under alkaline stress and saline-alkaline stress.

**Fig 9:**
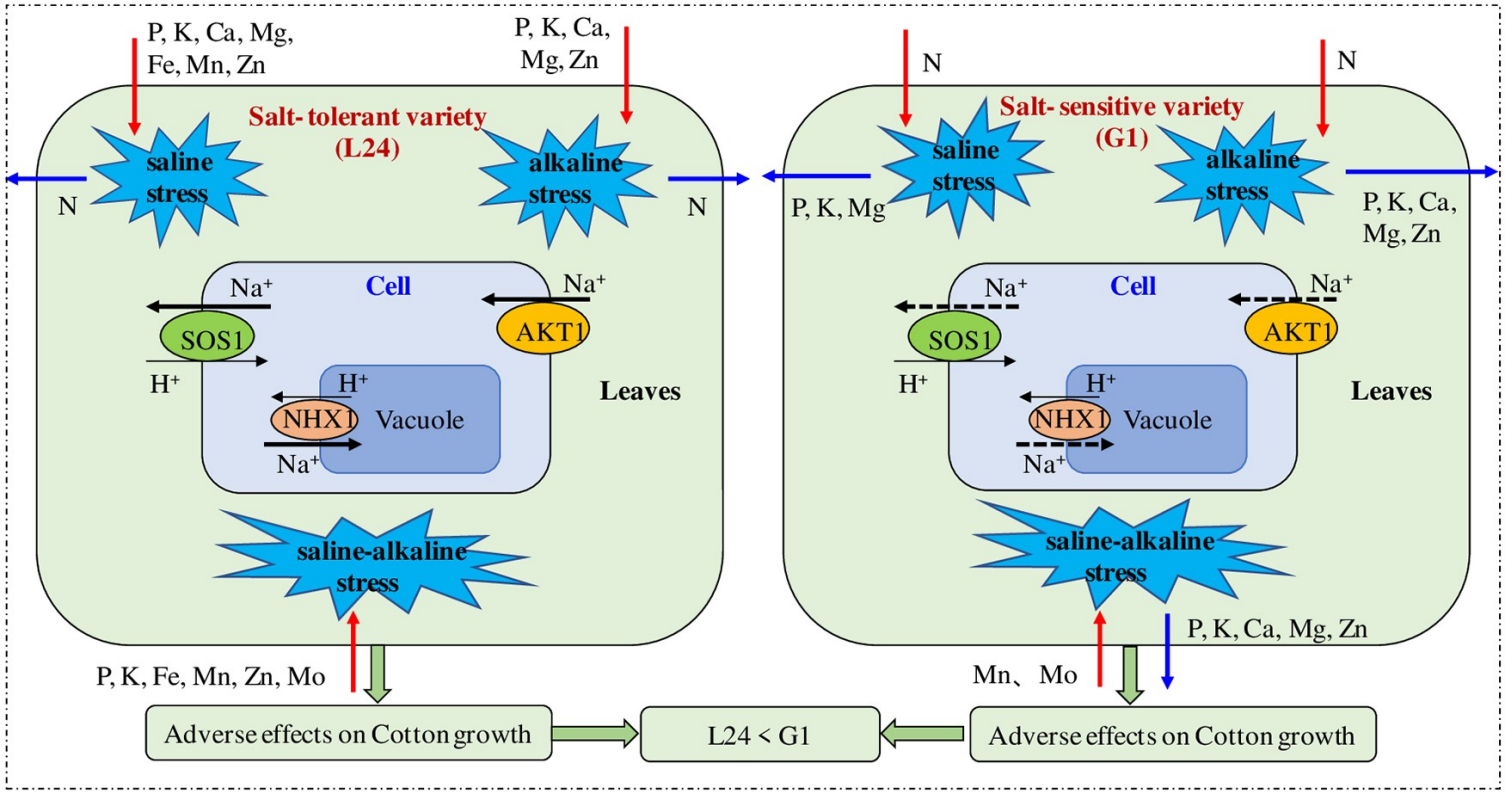
Response of ion content and gene expression in salt-tolerant variety and salt-sensitive variety to saline, alkaline and saline-alkaline stresses. The red arrow indicates the increase of ion content and the blue indicates the decrease; The black solid line indicates that the up-regulating of gene expression is relatively large, and the dotted line indicates that the up-regulating is relatively small.

Under salt stress, Na^+^ is the key ion affecting ion homeostasis in plants, so the main focus of this research was to study the transport mechanism of Na^+^ ions and their compartmentalization [23]. In the present study, we assessed the expression of Na^+^ transport-related genes such as *GhSOS*1, *GhNHX*1 and *GhAKT*1 in cotton. An increase in the relative expression of the *GhSOS*1 gene can promote Na^+^ efflux and reduce Na^+^ accumulation in cells [43]. Our results showed that the relative expression of the *GhSOS*1 gene in the two cotton varieties increased significantly with increasing soil salinity. Under alkaline stress and saline-alkaline stress, the relative expression of the *GhSOS*1 gene in the two cotton varieties first increased and then decreased with increasing stress level (Fig 8). This indicated that a high pH environment inhibited the expression of the *GhSOS*1 gene and resulted in the accumulation of Na^+^ in cells. In addition, we found that *GhSOS*1 expression in the salt-tolerant variety was significantly higher than that in the salt-sensitive variety under alkaline stress and saline-alkaline stress. Previous studies have also demonstrated that *GhSOS*1 overexpression enhanced the salt tolerance of cotton [43]. In recent years, predecessors have conducted substantial research on the NHX gene, and the results showed that overexpression of *GhNHX*1 can significantly enhance the salt tolerance of cotton [24, 49]. In our experiment, *GhNHX*1 expression in the salt-tolerant variety increased significantly under different salt stresses, and *GhNHX*1 expression was higher under saline stress and alkaline stress than under saline-alkaline stress. In contrast, the expression of *GhNHX*1 in the salt-sensitive variety after A2 and SA1 treatment was the same or lower than that in the CK group. Guo et al. [40] also found that saline stress significantly increased the relative expression of *GhNHX*1 in the roots and leaves of cotton, but alkaline stress had no significant effect on the relative *GhNHX*1 expression. In this study, *GhNHX*1 expression in the salt-tolerant variety was significantly higher than that in the salt-sensitive variety under saline, alkaline and saline-alkaline stresses. The overexpression of *GhNHX*1 in salt-tolerant varieties under salt stress could help to isolate excess Na^+^ and regulate ion homeostasis. The *GhAKT*1 gene is the main channel gene for K^+^ transport to the aerial parts through the root system [25]. AKT1 participates in salinity stress responses and plays crucial roles in the maintenance of K^+^/Na^+^ homeostasis under salt conditions [50]. We found that the variation in *GhAKT*1 expression in the two cotton varieties was similar to the expression of the *GhSOS*1 gene under different salt stresses. The expression of the *GhAKT*1 gene under saline-alkaline stress was significantly lower than that under saline stress or alkaline stress, indicating that the uptake and transport of K^+^ was more seriously inhibited. In addition, *GhAKT*1 expression in the salt-tolerant variety under alkaline stress and saline-alkaline stress was significantly higher than that in the salt-sensitive variety. Na^+^ transport-related gene expression analysis showed that different genotypes of cotton adapt to salt stress by up-regulating and down-regulating the expression of genes to re-establish ion homeostasis. Effects of different salt (saline, alkaline and saline-alkaline) stresses on ion content and gene expression in contrasting cotton varieties (salt-tolerant and salt-sensitive) was summarised as Fig 9. Our results may be helpful to clarify the salt tolerance mechanism of different genotypes of cotton.

## 5. Conclusions

Saline-alkaline stress had more serious adversely effect on growth of two cotton varieties (salt-tolerant and salt-sensitive) than saline stress or alkaline stress alone. Compared with saline stress, alkaline stress significantly inhibited root growth and reduced the root-to-shoot ratio of cotton. Under different salt stresses, the concentration of Na in cotton organs increased significantly, and saline-alkaline stress was the largest. The salt-tolerant variety had lower Na and higher K concentrations in the leaves, stems and roots than the salt-sensitive variety. Under saline, alkaline, and saline-alkaline stresses, most of the elements (except N) in the leaves of salt-tolerant variety increased, while salt-sensitive variety decreased. This indicated that salt-tolerant variety had strong ability of ion absorption and transport under salt stress. Moreover, the inhibition effect of alkaline and saline-alkaline stress on ion absorption and transport of salt-sensitive variety was greater than that of saline stress. The relative expressions of *GhSOS*1 and *GhAKT*1 in salt-sensitive variety under alkaline and saline-alkaline stress were also lower than those under saline stress. Compared with salt-sensitive variety, the relative expressions of *GhSOS*1, *GhNHX*1 and *GhAKT*1 in salt-tolerant variety significantly up-regulated under alkaline and saline-alkaline stress, which revealed that salt-tolerant cotton can promote Na^+^ efflux and compartmentalization of Na^+^ in vacuoles. Therefore, salt-tolerant cotton varieties have an internal mechanism to maintain ionic homeostasis.

## Supporting information

S1 Table Primers used for gene detection as well as qRT-PCR analysis. (DOC)

S2 Table Reverse transcription reaction system. (DOC)

S3 Table PCR transcription reaction system. (DOC)
