# Supplementary material for: Ion homeostasis and Na+ transport-related gene expression in two cotton (Gossypium hirsutum L.) varieties under saline, alkaline and saline-alkaline stresses
Source: PLoS One. 2021 Aug 10;16(8):e0256000. doi: 10.1371/journal.pone.0256000 (PMC8354432; doi:10.1371/journal.pone.0256000)
Supplement: S1 Table — (DOC) [file pone.0256000.s001.doc]

**S1 Table** Primers used for gene detection as well as qRT-PCR analysis

| Primer | Primer Sequences (5’-3’) F | Primer Sequences(5’-3’) R |
| --- | --- | --- |
| GAPDH-S/A | TGATGCCAAGGCTGGAATTGCTT | GTGTCGGATCAAGTCGATAACACGG |
| SOS1-S/A | AAGTCAGGTTCTACAACAGCCAG | CCTTCAAGTGTTGAAATATCAAAT |
| AKT1-S/A | CCTCGGAAGGTTTACAAGCGA | TACTGCTCTTACGCCTCGGTC |
| NHX1-S/A | TTCTCTTTCTTTATGTCGGGATG | AACAAGACCCATCAGCACAGC |
| DFR-S/A | TAATGTTCCCACCAAGTTCAA | AAACTCAAATCCCAAGTCCAA |
