# Supplementary material for: Ion homeostasis and Na+ transport-related gene expression in two cotton (Gossypium hirsutum L.) varieties under saline, alkaline and saline-alkaline stresses
Source: PLoS One. 2021 Aug 10;16(8):e0256000. doi: 10.1371/journal.pone.0256000 (PMC8354432; doi:10.1371/journal.pone.0256000)
Supplement: S2 Table — (DOC) [file pone.0256000.s002.doc]

**S2 Table** Reverse transcription reaction system

| Composition | Volume |
| --- | --- |
| RNA | 2 μg |
| Oligo(dT) (50 uM) | 1 μl |
| dNTP Mix(10 mmol/L) | 1 μl |
| 5×Reaction Buffer | 4 μl |
| RNase Inhibitor (40 U/μl) | 0.5 μl |
| MMLV RT (200 U/μl) | 1 μl |
| RNase free dH2O | Up to 20 μl |
