# Supplementary material for: Ion homeostasis and Na+ transport-related gene expression in two cotton (Gossypium hirsutum L.) varieties under saline, alkaline and saline-alkaline stresses
Source: PLoS One. 2021 Aug 10;16(8):e0256000. doi: 10.1371/journal.pone.0256000 (PMC8354432; doi:10.1371/journal.pone.0256000)
Supplement: S3 Table — (DOC) [file pone.0256000.s003.doc]

**S3 Table** PCR transcription reaction system

| Composition | Volume |
| --- | --- |
| 2×SYBR real-time PCR premixture | 10 |
| primer | 0.8 |
| cDNA | 1 |
| ddH2O | 8.2 |
